# Retraction: Safety evaluation of antituberculosis drugs during pregnancy: a systematic review and meta-analysis

**DOI:** 10.3389/fsurg.2024.1540233

**Published:** 2024-12-18

**Authors:** Frontiers Editorial Office

A Retraction of the Systematic Review Safety evaluation of antituberculosis drugs during pregnancy: a systematic review and meta-analysis By Zhou X, Fang G, Xie Y, Wei A and Huang F (2022). Front. Surg. 9. doi: 10.3389/fsurg.2022.871321

Following publication, concerns were raised regarding the scientific validity of the article. An investigation was conducted in accordance with Frontiers’ policies.

It was found that the concerns were valid and that the article does not meet the standards of editorial and scientific soundness for Frontiers in Surgery; therefore, the article has been retracted.

The retraction was approved by the Chief Editors for Frontiers in Surgery and the Chief Executive Editor of Frontiers. The authors do not approve of the retraction.

